# Angiotensin II AT_**1**_ Receptors Are Involved in Neuronal Activation Induced by Amphetamine in a Two-Injection Protocol

**DOI:** 10.1155/2013/534817

**Published:** 2013-09-08

**Authors:** Maria Constanza Paz, Natalia Andrea Marchese, Liliana M. Cancela, Claudia Bregonzio

**Affiliations:** Departamento de Farmacología, Facultad de Ciencias Químicas Universidad Nacional de Córdoba, Instituto de Farmacología Experimental Córdoba (IFEC-CONICET), 5000 Córdoba, Argentina

## Abstract

It was already found that Ang II AT_1_ receptors are involved in the neuroadaptative changes induced by a single exposure to amphetamine, and such changes are related to the development of behavioral and neurochemical sensitization. The induction of the immediately early gene c-fos has been used to define brain activated areas by amphetamine. Our aim was to evaluate the participation of AT_1_ receptors in the neuronal activation induced by amphetamine sensitization. The study examined the c-fos expression in mesocorticolimbic areas induced by amphetamine challenge (0.5 mg/kg i.p) in animals pretreated with candesartan, a selective AT_1_ receptor blocker (3 mg/kg p.o × 5 days), and amphetamine (5 mg/kg i.p) 3 weeks before the challenge. Increased c-fos immunoreactivity was found in response to the amphetamine challenge in the dorsomedial caudate-putamen and nucleus accumbens, and both responses were blunted by the AT_1_ receptor blocker pretreatment. In the infralimbic prefrontal cortex, increased c-fos immunoreactivity was found in response to amphetamine and saline challenge, and both were prevented by the AT_1_ receptor blocker. No differences were found neither in ventral tegmental area nor prelimbic cortex between groups. Our results indicate an important role for brain Ang II in the behavioral and neuronal sensitization induced by amphetamine.

## 1. Introduction

It has been demonstrated that the enhanced responses to psychostimulants rely on time-dependent neuroadaptations that involves enduring alterations in behavioral and neurochemical responses. These changes, known as sensitization, are associated with long-lasting hyperreactivity of the central dopaminergic mesolimbic pathway [1–3]. Repeated exposure is not necessary, since a single exposure to psychostimulants or even morphine is sufficient to induce persistent locomotor sensitization and neurochemical and electrophysiological changes in rodents [4–6]. In the two-injection protocol, changes in responsiveness are induced by the first psychostimulant administration and are revealed by the second one. This model is a very simple paradigm and is more sensitive than the repeated injection protocol to study the bases of long-term effects of drugs of abuse [5].

One of the principal systems involved in drug abuse is the dopaminergic mesolimbic system, a critical component in the brain reward circuit [7]. Brain Angiotensin II (Ang II) belongs to the group of peptides known to stimulate dopamine release [8–10]. Moreover, the Ang II receptors are located in dopamine-rich brain areas [10, 11], such as the nucleus accumbens (Nacc) and caudate-putamen (CPu), strongly related to self-administration and sensitization to drugs of abuse [12].

Sensitization to drugs such as amphetamine is associated with alterations in the morphology of neurons in the Nacc, a brain region critical to motivation and reward. It is known that Ang II is involved in the control of salt appetite, and the sodium depletion that promotes salt appetite also leads to alterations in neurons in the Nacc. In this sense, the medium spiny neurons within the shell of the Nacc of rats that had experienced sodium depletions had significantly more dendritic branches and spines than controls [13]. In addition, a history of sodium depletion was found to have cross-sensitization effects, leading to enhanced psychostimulant responses to amphetamine [14]. Thus, common neuroadaptations in response to salt and amphetamine may provide a general mechanism for the enhanced responses induced by reexposure to these challenges.

In a previous work, we showed that Ang II AT_1_ receptors were involved in the neuroadaptative changes induced by a single exposure to amphetamine and that such changes were related to the development of behavioral and neurochemical sensitization [6].

The induction of immediately early gene c-fos is a well-accepted marker of neuronal activation, and this approach has been used to define areas involved in the actions induced by amphetamine, since enhanced c-fos expression in the CPu, Nacc, prefrontal cortex (PFC), and ventral tegmental area (VTA) was found after amphetamine administration [15].

In order to extend our previous findings, we evaluated the participation of AT_1_ receptors in the neuronal activation induced by amphetamine sensitization in the nucleus accumbens core and shell (Nacc core, Nacc shell), CPu, PFC, and VTA. Experiments were performed using the same protocols and doses of AT_1_ receptor blocker and amphetamine in a context-independent manner previously used [6]. Although the contextual environment strengthens the expression of sensitized responses when tested in the context previously associated with drug administration [16], it has been shown that amphetamine induced sensitization with the two-injection protocol in both context-dependent and in context-independent manner, while cocaine induced sensitization in context-dependent manner [5]. In the present study, the amphetamine administrations were performed in different environments to induce responses in context-independent conditions. To our knowledge, this is the first study that aims to analyze the involvement of brain Ang II in altered neuronal activation using the two-injection protocol of amphetamine administration in a context-independent manner.

## 2. Materials and Methods

### 2.1. Animals

Adult male Wistar rats (250–330 g), purchased from the Facultad de Ciencias Veterinarias (Universidad Nacional de La Plata, Buenos Aires, Argentina), were used in this study. Animals were maintained at 20–24°C under a 12 h light-dark cycle (lights on at 0700 hours) with free access to food and water. Upon arrival, they were placed in the colony room for at least 7 days before experimental tests and randomly housed in groups of four per cage. All procedures were approved by the Animal Care and Use Committee of the Facultad de Ciencias Químicas, Universidad Nacional de Córdoba, Argentina, in accordance with the NIH Guide for the Care and Use of Laboratory Animals. 

### 2.2. Experimental Procedure

The animals received candesartan cilexetil 3 mg/kg or vehicle by oral administration (intragastric using a feeding needle) once a day during 5 days. Twenty-four hours after the last administration, they were injected with D-amphetamine 5 mg/kg or saline i.p. A total of 33 animals were assigned randomly to the treatments: vehicle-saline (veh-sal), vehicle-amphetamine (veh-amph), candesartan-saline (cv-sal), and candesartan-amphetamine (cv-amph), and they were left undisturbed in their home cages until the day of the experiment. Twenty-one days after pretreatment, amphetamine-induced neuronal activation was evaluated in the four groups using a challenge injection of amphetamine 0.5 mg/kg i.p. This second injection of the psychostimulant was given in a different environment to avoid context association with the drug. To discriminate the response to amphetamine challenge, another group of 20 animals were assigned randomly to the same four treatments and tested after a challenge injection of saline. 

### 2.3. Staining Procedure for Fos and Fos/Tyrosine Hydroxylase (TH) Immunohistochemistry

Fos immunoreactivity assay was performed in Nacc core, Nacc shell, CPu, PFC, and VTA. The double-labeling with Fos/TH antibodies was performed only in VTA in order to identify differential activation in catecholaminergic neurons since dopaminergic neurons in this nucleus send projections to Nacc and prefrontal cortex.

Ninety minutes after the amphetamine or saline challenge injection, the animals were prepared for brain fixation for immunohistochemical detection of Fos or Fos and TH. This approach was selected because the increased formation of Fos protein, 1 to 2 h after stimulation, correlates with increased neural activity in a wide range of neural systems [17, 18]. The animals were anesthetized with chloral hydrate 16% (400 mg/kg i.p.) and perfused transcardially with 250 mL of normal saline and heparine (200 *μ*L/L), followed by 400 mL of 4% paraformaldehyde in 0.1 M phosphate buffer (PB, pH 7.4). The brains were removed, fixed in the same solution overnight, and then stored at 4°C in PB containing 30% sucrose. Coronal sections of 40 *μ*m were cut using a freezing microtome (Leica CM15105) and collected in PB 0.01 M. They were placed in a mixture of 10% H_2_O_2_ and 10% methanol until oxygen bubbles ceased appearing. Samples were incubated in 10% normal horse serum (NHS) (GIBCO, Auckland, NZ) in PB for 2 h to block nonspecific binding sites. 

All of the free-floating sections from each brain were first processed for Fos immunoreactivity (Fos-IR) using an avidin biotin-peroxidase procedure. Sections of the VTA were then stained for TH immunoreactivity (TH-IR).

The staining procedures followed the double-labeling procedures described in Franchini and Vivas [19] and Franchini et al. [20]. Briefly, the free-floating sections were incubated overnight at room temperature in a rabbit anti-fos antibody (produced in rabbits against a synthetic 14-amino acid sequence, corresponding to residues 4–17 of human Fos) (Ab-5; Oncogene Science, Manhasset, NY), diluted 1 : 20,000 in PB containing 2% NHS and 0.3% Triton X-100 (Flucka Analytical). The sections were then rinsed with PB 0.01 M and incubated with biotin-labeled universal secondary antibody (diluted 1 : 2,000 in 2% NHS-PB) and avidin-biotin-peroxidase complex (Vector Laboratories, Burlingame, CA; diluted 1 : 200 in 2% NHS-PB), for 2 h each at room temperature. The peroxidase label was detected with diaminobenzidine hydrochloride (Sigma Chemical Co.); the solution was intensified with 1% cobalt chloride and 1% nickel ammonium sulfate. This method produces a violet nuclear reaction product. 

The Fos-labeled sections, also processed for immunocytochemical localization of TH, were rinsed and incubated in 10% NHS in PB for 2 h. Immediately after, they were incubated for 48 h at 4°C with monoclonal TH antibody (Millipore, Tecnolab S.A., diluted 1 : 5,000 in PB with 2% NHS and 0.3% Triton X-100). After incubation, the sections were rinsed and incubated with biotin-labeled mouse secondary antibody (Jackson Laboratories (P) Ltd., diluted 1 : 5,000 in 2% NHS-PB) and avidin-biotin-peroxidase complex, for 2 h each at room temperature. Cytoplasmic TH-IR was detected by unintensified diaminobenzidine hydrochloride, which produces a brown reaction product. Finally, the free-floating sections were mounted on gelatinized slides, air-dried overnight, dehydrated, cleared in xylene, and placed under a coverslip with DPX mountant for histology (Flucka Analytical). 

### 2.4. Cytoarchitectural and Quantitative Analysis

Images containing Fos-IR nuclei were obtained by using a computerized system that included a Leica DM 4000 B microscope equipped with a DFC Leica digital camera attached to a contrast enhancement device. The brain nuclei evidencing Fos-IR were identified and delimited according to atlas of Paxinos [21]. The Fos-IR nuclear profiles were counted at the dorsomedial region of CPu (corresponding to plates with a distance of 2.28 mm to 1.92 mm from bregma), Nacc core and shell (corresponding to plates with a distance of 2.28 mm to 1.92 mm from bregma), PFC (corresponding to plates with a distance of 3.72 mm to 3.00 mm from bregma), and VTA (corresponding to plates with a distance of −6.24 mm to −6.48 mm from bregma). The brain sections were processed concurrently for subjects across all groups. Images were standardized using Adobe Photoshop image analysis program (version 5.5). Counting of Fos-IR was accomplished using IMAGE J software from the National Institutes of Health (NIH). Threshold was fixed between an interval of 0 and 150 in black and white conditions, and all higher values were considered background. Fos-IR neurons were identified by dense black staining of the nucleus and counted by setting a size range for cellular nuclei (8 to 12 *μ*m of diameter). 

The measurement for each brain area was done bilaterally in two sections. The value obtained was the average of the four countings. The counting was made on a 0.37 mm^2^ area (corresponding to 200x magnification) in animals of each condition for amphetamine challenge (5–8 animals) and for saline challenge (4-5 animals). Because the size and section thickness of nuclei did not change between experimental and control groups, any systematic error should be identical for all groups. Hence, the results are meant to provide relative data on expression of Fos-IR but are not meant to be accurate estimates of absolute cell counts. The counting was made by two operators on each section analyzed, to ensure that the number of profiles obtained was similar, but only one counting was used. Counting of Fos-IR cells was performed blinded to the experimental groups.

### 2.5. Double Immunostaining Fos/TH Quantification

Fos-IR nuclei (violet) were identified and counted only in positive TH cells labeled by brown. The counting was performed in VTA in two sections (corresponding to plates with a distance of −6.24 to −6.48 mm from bregma) under microscope.

### 2.6. Statistical Analysis

Data were reported as means ± SEM. The study design used three-way ANOVA with vehicle and candesartan as pretreatment factor; saline and amphetamine as treatment factor; and challenge with saline or amphetamine as the third factor analyzed. If an interaction and/or main effect was observed, pair-wise comparisons following ANOVA were made using the Bonferroni post test. A value of *P* < 0.05 was considered significant. The analysis was performed using STATISTICA 7 software.

## 3. Results

### 3.1. Distribution of Fos in the Dorsomedial Region of Caudate Putamen, Nucleus Accumbens Core and Shell

**Figure 1:**
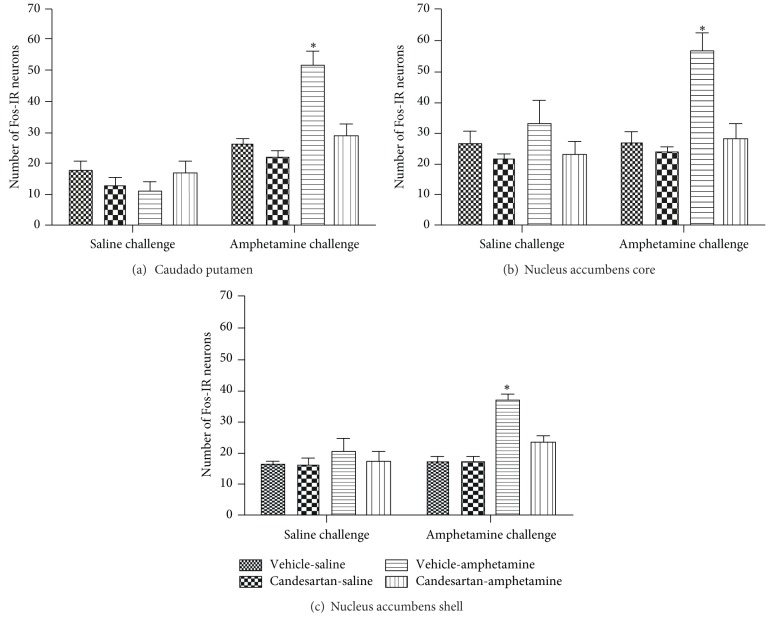
Average number of Fos-IR neurons in brain nuclei: dorsomedial region of CPu (a), Nacc core (b), and Nacc shell (c), in response to a challenge of saline (*n* = 4-5) or amphetamine (*n* = 5–8), 21 days after a pretreatment with cv or veh and a treatment with amph or sal (veh-sal, cv-sal, veh-amph, and cv-amph). Values are means ± SEM. *P < 0.05 significantly different from the other amphetamine-challenged groups (3-way ANOVA, post hoc Bonferroni).

As shown in Figure 1, the amphetamine challenge induced a significant increase in the number of Fos-IR cells in vehicle-amphetamine rats compared to controls (vehicle-saline and candesartan-saline groups) within three areas of analysis: CPu (Figure 1(a)), Nacc core (Figure 1(b)), and Nacc shell (Figure 1(c)). Pretreatment with candesartan significantly prevented the increase in number of Fos-IR cells in amphetamine-treated rats within these three areas.

CPu: the results obtained from the three-way ANOVA analysis were PRETREATMENT *F*_(1,39)_ = 6.69,  *P* < 0.01, TREATMENT *F*_(1,39)_ = 8.45,  *P* < 0.006, CHALLENGE *F*_(1,39)_ = 43.55,  *P* < 0.000001, INTERACTION pretreatment ∗ challenge *F*_(1,39)_ = 6.35,  *P* < 0.02, INTERACTION treatment ∗ challenge *F*_(1,39)_ = 11.58,  *P* < 0.002, and INTERACTION pretreatment ∗ treatment ∗ challenge *F*_(1,39)_ = 8.17,  *P* < 0.007. Bonferroni post hoc comparisons indicated that the veh-amph group was significantly different from the veh-sal (*P* < 0.00005), cv-sal (*P* < 0.000001), and the cv-amph groups (*P* < 0.0003) after amphetamine challenge. Representative microphotographs are shown in Figure 3 (C_1_ to F_1_). Saline challenge did not produce significant differences in the Fos-IR cells pattern between treatments in this area (Figure 1(a)).

Nacc core: the results obtained from the three-way ANOVA analysis were PRETREATMENT *F*_(1,39)_ = 12.28,  *P* < 0.001, TREATMENT *F*_(1,39)_ = 10.14,  *P* < 0.003, CHALLENGE *F*_(1,39)_ = 5.36,  *P* < 0.03, and INTERACTION pretreatment ∗ treatment *F*_(1,39)_ = 5.07,  *P* < 0.03. Bonferroni post hoc comparisons indicated that the veh-amph group was significantly different from the veh-sal (*P* < 0.0006), cv-sal (*P* < 0.00003) and from the cv-amph groups (*P* < 0.0004) after amphetamine challenge. Representative microphotographs are shown in Figure 3 (C_2_ to F_2_). Saline challenge did not produce significant differences in the Fos-IR cells pattern between treatments in this area (Figure 1(b)).

Nacc shell: the results obtained from the three-way ANOVA analysis were PRETREATMENT *F*_(1,39)_ = 7.16,  *P* < 0.01, TREATMENT *F*_(1,39)_ = 17.58,  *P* < 0.0002, CHALLENGE *F*_(1,39)_ = 10.07,  *P* < 0.003, INTERACTION pretreatment ∗ treatment *F*_(1,39)_ = 6.79,  *P* < 0.01, and INTERACTION treatment ∗ challenge *F*_(1,39)_ = 7.61,  *P* < 0.009. Bonferroni post hoc comparisons indicated that the veh-amph group was significantly different from the veh-sal (*P* < 0.000007), cv-sal (*P* < 0.000006), and the cv-amph groups (*P* < 0.0003) after amphetamine challenge. Representative microphotographs are shown in Figure 3 (C_3_ to F_3_). Saline challenge did not produce significant differences in the Fos-IR cells pattern between treatments in this area (Figure 1(c)).

### 3.2. Distribution of Fos in the Infralimbic and Prelimbic Cortex

**Figure 2:**
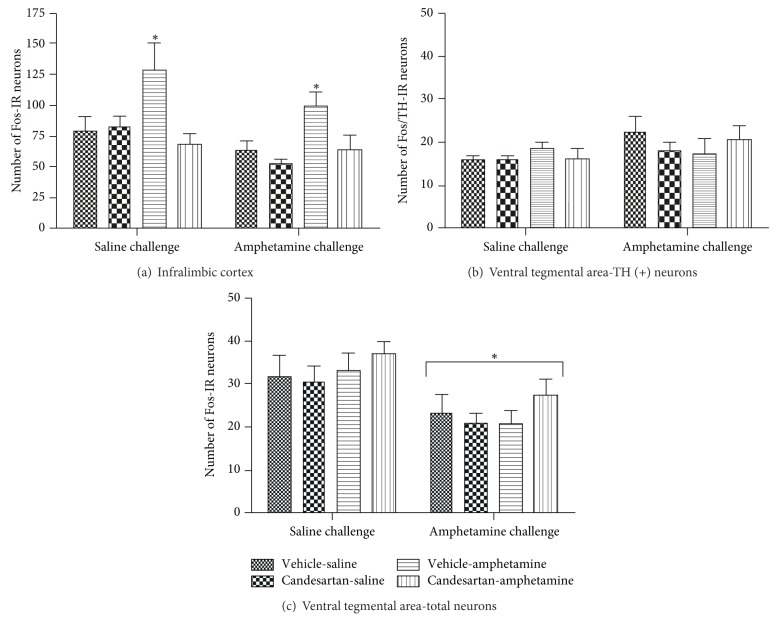
Average number of Fos-IR neurons in brain nuclei: IL cortex (a), VTA (c), in response to a challenge of saline (*n* = 4-5) or amphetamine (*n* = 5–8), 21 days after a pretreatment with cv or veh and a treatment with amph or sal (veh-sal, cv-sal, veh-amph, and cv-amph). Values are means ± SEM. *P < 0.05 significantly different from the other groups in response to the same challenge (3-way ANOVA, post hoc Bonferroni). Average number of Fos/TH-IR neurons in VTA (b), in response to a challenge of saline (*n* = 4-5) or amphetamine (*n* = 5–8), 21 days after a pretreatment with cv or veh and a treatment with amph or sal (veh-sal, cv-sal, veh-amph and cv-amph). Values are means ± SEM. *P < 0.05 significantly different from the salin-challenged groups (3-way ANOVA, post hoc Bonferroni).

**Figure 3:**
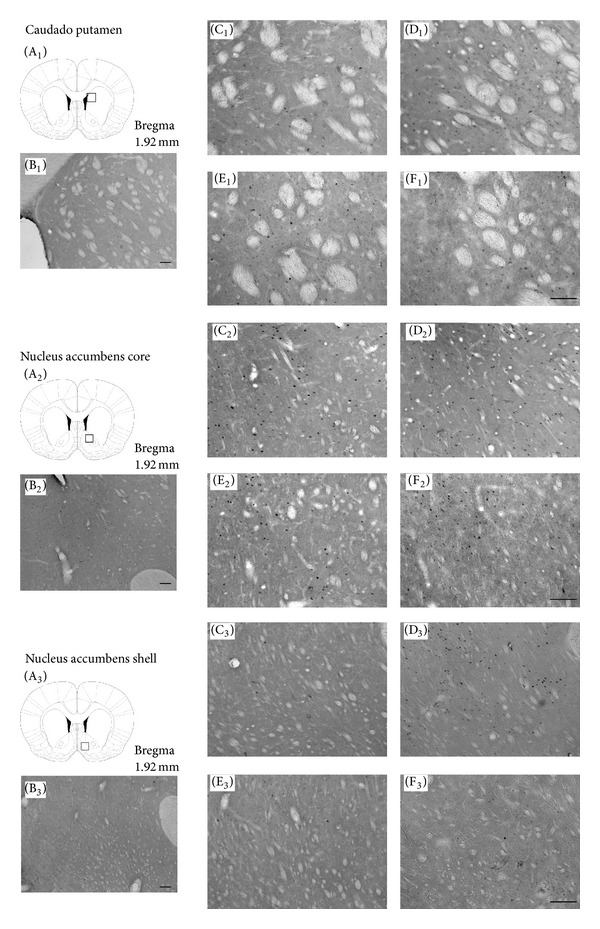
(A) Schematic coronal slice indicating the region where the counting was done in different areas: CPu (A_1_), Nacc core (A_2_), and Nacc shell (A_3_). (B) Photomicrographs 100x magnification: CPu (B_1_), Nacc core (B_2_), and Nacc shell (B_3_). Photomicrographs 200x magnification showing the pattern of Fos-IR neurons after an amphetamine challenge in (C) veh-sal group in different areas: CPu (C_1_), Nacc core (C_2_), and Nacc shell (C_3_); (D) veh-amph group in CPu (D_1_), Nacc core (D_2_), and Nacc shell (D_3_); (E) cv-sal group in CPu (E_1_), Nacc core (E_2_), and Nacc shell (E_3_); and (F) cv-amph group in CPu (F_1_), Nacc core (F_2_), and Nacc shell (F_3_). Scale bar = 100 *μ*m.

**Figure 4:**
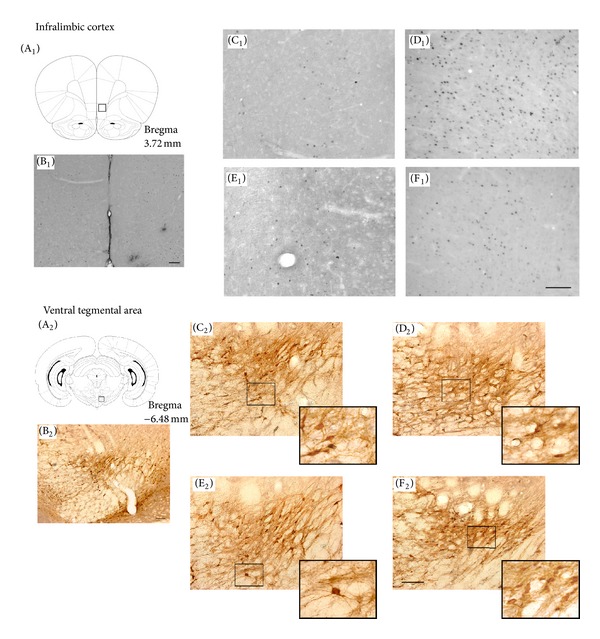
(A) Schematic coronal slice indicating the region where the counting was done in different areas: IL cortex (A_1_), VTA (A_2_). (B) Photomicrographs 100x magnification: IL cortex (B_1_) and VTA (B_2_). Photomicrographs 200x magnification of the pattern of Fos-IR neurons in IL cortex or Fos/TH-IR neurons in VTA after an amphetamine challenge in (C) veh-sal group in different areas: IL cortex (C_1_), VTA (C_2_); (D) veh-amph group in IL cortex (D_1_), VTA (D_2_); (E) cv-sal group in IL cortex (E_1_), VTA (E_2_); and (F) cv-amph group in IL cortex (F_1_), VTA (F_2_). A section of each photomicrographs of Fos/TH-IR (indicated by a square) is shown in a higher magnification (1,000x). Scale bar = 100 *μ*m.

Infralimbic cortex (IL): as shown in Figure 2(a), saline and amphetamine challenges induced a significant increase in the number of Fos-IR cells in vehicle-amphetamine rats compared to controls (vehicle-saline and candesartan-saline groups). Pretreatment with candesartan significantly prevented the increase in number of Fos-IR cells in amphetamine-treated rats within this brain area. The results obtained from the three-way ANOVA analysis were PRETREATMENT *F*_(1,28)_ = 5.51,  *P* < 0.03, TREATMENT *F*_(1,28)_ = 11.83,  *P* < 0.002, CHALLENGE *F*_(1,28)_ = 1.53,  *P* = 0.22, and INTERACTION pretreatment ∗ treatment *F*_(1,28)_ = 5.40,  *P* < 0.03. Bonferroni post hoc comparisons indicated that the veh-amph group was significantly different from the veh-sal (*P* < 0.003), cv-sal (*P* < 0.001), and the cv-amph groups (*P* < 0.01) independently of the challenge received. Representative microphotographs are shown in Figure 4 (C_1_ to F_1_).

Prelimbic cortex: no significant differences between groups were found in this region of the prefrontal cortex. The results obtained from the three-way ANOVA analysis were PRETREATMENT *F*_(1,34)_ = 0.91,  *P* = 0.35, TREATMENT *F*_(1,34)_ = 3.30,  *P* = 0.08, and CHALLENGE *F*_(1,34)_ = 6.75,  *P* < 0.01, and no interaction between factors were found. Bonferroni post hoc comparison indicated that amphetamine challenged groups were significantly different from saline challenged groups (*P* < 0.02). Data not shown.

### 3.3. Distribution of Fos and Fos/TH in the Ventral Tegmental Area

No differences were found in the number of Fos/TH-IR double-labeled (Figure 2(b)) and Fos-IR (Figure 2(c)) neurons at VTA, following neither saline nor amphetamine challenge. Fos/TH-IR: amphetamine challenge did not produce differences in the activation pattern of VTA neurons in vehicle-amphetamine rats compared to controls (vehicle-saline and candesartan-saline groups). No significant differences were observed between vehicle-amphetamine and candesartan-amphetamine groups. Representative microphotographs are shown in Figure 4 (C_2_ to F_2_). Saline challenge did not produce significant differences in the Fos/TH-IR cells pattern between treatments in VTA (Figure 2(b)). 

The results obtained from the three-way ANOVA analysis were PRETREATMENT *F*_(1,36)_ = 0.01,  *P* = 0.91, TREATMENT *F*_(1,36)_ = 0.14,  *P* = 0.71, and CHALLENGE *F*_(1,36)_ = 1.30,  *P* = 0.26.

Fos-IR: amphetamine challenge did not produce differences in the activation pattern of VTA neurons in vehicle-amphetamine rats compared to controls (vehicle-saline and candesartan-saline groups). No significant differences were observed between vehicle-amphetamine and candesartan-amphetamine groups. Representative microphotographs are shown in Figure 4 (C_2_ to F_2_). Saline challenge did not produce significant differences in the Fos-IR cells pattern between groups in VTA (Figure 2(c)).

The results obtained from the three-way ANOVA analysis were PRETREATMENT *F*_(1,36)_ = 0.37,  *P* = 0.54, TREATMENT *F*_(1,36)_ = 1.25,  *P* = 0.27, CHALLENGE *F*_(1,36)_ = 14.07,  *P* < 0.0006. Bonferroni post hoc comparison indicated that amphetamine challenged groups were significantly different from saline challenged groups (*P* < 0.0006). 

## 4. Discussion

The main finding of this work is that Ang II AT_1_ receptors are involved in the long-lasting neurochemical adaptations induced by a single exposure to amphetamine. These results are in agreement with previous findings showing the involvement of Ang II AT_1_ receptors in behavioral sensitization using the same protocol of amphetamine treatment in a context-independent manner [6]. To our knowledge, this is the first study performed using the two-injection protocol of amphetamine administration in a context-independent manner that aims to analyze the involvement of brain Ang II in the altered neuronal activation induced by amphetamine exposure.

Considering that repeated exposure to psychostimulants is not necessary for development of sensitization, since a single exposure is sufficient to induce persistent locomotor sensitization and neurochemical changes, in this study we used the two-injection protocol of amphetamine. Moreover, the protocols used for repeated drugs of abuse administration elicit different biochemical and cellular responses than single exposures, which could hamper the subsequent interpretation of causality links [5]. Then, with the protocol of amphetamine administration used in the present work, the first injection induces the changes in responsiveness and the second injection unmasks the existence of neuroadaptations [5, 6]. 

Behavioral sensitization is not limited to addictive drugs, and it can also be induced by strong motivational or affective states (thirst or hunger) associated with natural rewards (water, salt, food, etc.) [22]. Recently, behavioral cross-sensitization between sodium depletion and cocaine has also been described [23]. The results from these experiments indicate that treatments generating a sustained salt appetite and producing cocaine-induced psychomotor responses show reciprocal behavioral cross-sensitization, similar to results found using amphetamine [14]. In relation to the brain renin-angiotensin system, it has been found that intracerebral administration of Ang II induces sensitization to its vasoactive effects, such as hypertension sensitization, and also it could be involved in the development of neuroadaptive changes related to behavioral sensitization induced by natural reinforces and drugs of abuse [22]. Moreover, the sensitization of sodium appetite and thirst has been associated with central actions of Ang II and Aldosterone [24]. 

The induction of immediate early genes, like c-fos, is a well-accepted marker of neuronal activation, and it has been used to define brain areas putatively involved in the actions of amphetamine. Furthermore, dissociation between c-fos induction and neuronal electrophysiological activation has also been described [15]. Amphetamine-induced c-fos expression is quite high in brain areas receiving dopaminergic innervations, such as the CPu and Nacc. Rotllant et al. found a dose-dependent activation in the Nacc shell and all divisions of the paraventricular nucleus (PVN) and the amygdala [15]. Moreover, alterations in cellular activity associated with the expression of amphetamine sensitization in the CPu and Nacc have been described [7, 18]. Moreover, increased Fos expression in the Nacc core and shell has been described in animals with sodium depletion submitted to a sham-drinking paradigm, in which the persistent appetitive behavior and prolonged ingestion are similar to the behavior of animals responding to drugs of abuse [25].

In the present study, altered neuronal activation was found in response to the amphetamine challenge in limbic regions involved in the long-lasting changes associated with behavioral sensitization [5, 6, 18]. The increased Fos immunoreactivity induced by amphetamine challenge in the CPu, Nacc core, and Nacc shell, in animals previously exposed to amphetamine, was blunted by the AT_1_ receptor antagonist administration. These findings indicate a key role for brain Ang II in the development of the altered neuronal activation induced by amphetamine exposure.

Dopaminergic neurotransmission in the Nacc and CPu plays a critical role in the behavioral effects of psychostimulant drugs, and a close relationship has been shown between Ang II and dopaminergic neurotransmission in the brain. Functional interactions have also been described that correlate with anatomical findings showing high AT_1_ receptor density in dopamine-rich regions such as the CPu, hypothalamus, and Nacc [26, 27]. Chronic and single drug administration leads to behavioral sensitization [28, 29], and this phenomenon is associated with the sensitized ability to release dopamine in the Nacc and CPu [6, 29, 30]. We recently found that AT_1_ receptor blockade blunted the increased dopaminergic reactivity induced by amphetamine in the CPu and Nacc, showing a strong influence of Ang II in the development of this neuroadaptation [6]. Previous in vivo and in vitro studies support the assumption of a presynaptic localization of this receptor subtype [8, 26, 31]. Ang II AT_1_ receptor activation may be involved in the mechanisms that underlie the development of increased dopaminergic reactivity induced by amphetamine, which could be related to the altered neuronal activation in the CPu and Nacc observed in the present study. 

In the PFC, differences in Fos expression in the prelimbic region were not found. Meanwhile, differences in cellular activation were found in the infralimbic region, with a similar response to both challenges (saline and amphetamine) in animals previously exposed to amphetamine. In accordance with these results, previous studies have found an increased Fos expression in the medial prefrontal cortex, including the prelimbic and infralimbic regions, in cocaine-sensitized rats challenged two days after withdrawal [32]. A possible explanation for the increased neuronal activation to both challenges in the infralimbic region could be a sensitized response to stress and not to the psychostimulant, since there is considerable evidence of cross-sensitization between drugs of abuse and stress [33]. Interestingly, the AT_1_ receptor blocker prevented this response, corroborating the key role for Ang II in the stress response extensively described using AT_1_ receptor antagonists [34–38]. 

The cellular activation was also analyzed in catecholaminergic neurons in the VTA, and no differences between groups in response to amphetamine challenge were observed. Acute amphetamine induced dopamine release in the soma of dopaminergic neurons; however, Fos expression induced by acute amphetamine administration (1.5 and 5 mg/kg) was found mostly in GABAergic neurons and very little number in monoaminergic neurons [15]. It was also found that chronic amphetamine and cocaine administration lead to an accumulation of ΔfosB only in GABAergic neurons in the VTA, and according to Perroti et al., ΔfosB accumulation occurs only in neurons showing c-fos expression after acute drug administration [39]. This in agreement with the evidence supporting a key role for dopaminergic neurons in VTA in the induction of sensitization; meanwhile, the expression is associated with neuroadaptations in CPu and Nacc [1, 3]. When the total Fos immunoreactivity was analyzed in VTA, there was found a decrease in Fos expression in response to amphetamine challenge with respect to saline challenge independently of the animal's treatments (AT_1_ receptor blocker or amphetamine). 

The results in Fos expression obtained in VTA and prelimbic cortex, showing no effect to pretreatment and treatment, give a more specific role to the neuronal activation in the amphetamine-induced neuroadaptative responses in CPu and Nacc observed in the present study. 

In conclusion, these results taken together with our previous findings indicate that the development of behavioral sensitization and altered neuronal activation induced by amphetamine involve the AT_1_ receptor activation. Since the behavioral sensitization has been suggested as an adaptive process in addiction to psychostimulant and other drugs of abuse, it would be important to explore the brain AT_1_ receptors as a therapeutic target in drug abuse-related disorders. 

## Supplementary Material

Representative microphotographs showing no differences after saline challenge in the Fos-IR cell pattern in CPu, Nacc core, Nacc shell, IL cortex and VTA.
