# Supplementary material for: Angiotensin II AT1 Receptors Are Involved in Neuronal Activation Induced by Amphetamine in a Two-Injection Protocol
Source: Biomed Res Int. 2013 Sep 8;2013:534817. doi: 10.1155/2013/534817 (PMC3780567; doi:10.1155/2013/534817)

Supplementary figure 1

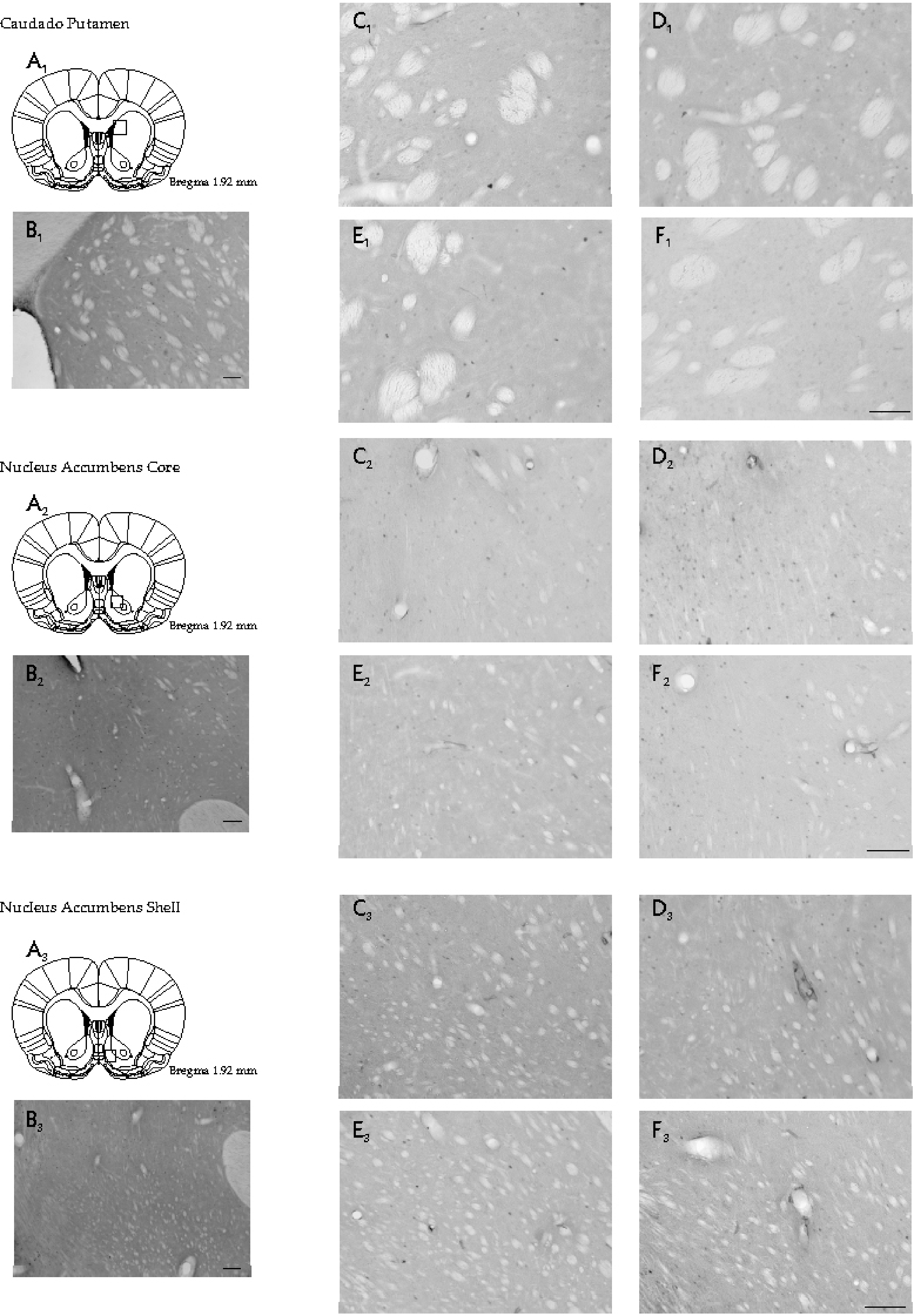

Supplementary figure 2

Infralimbic Cortex

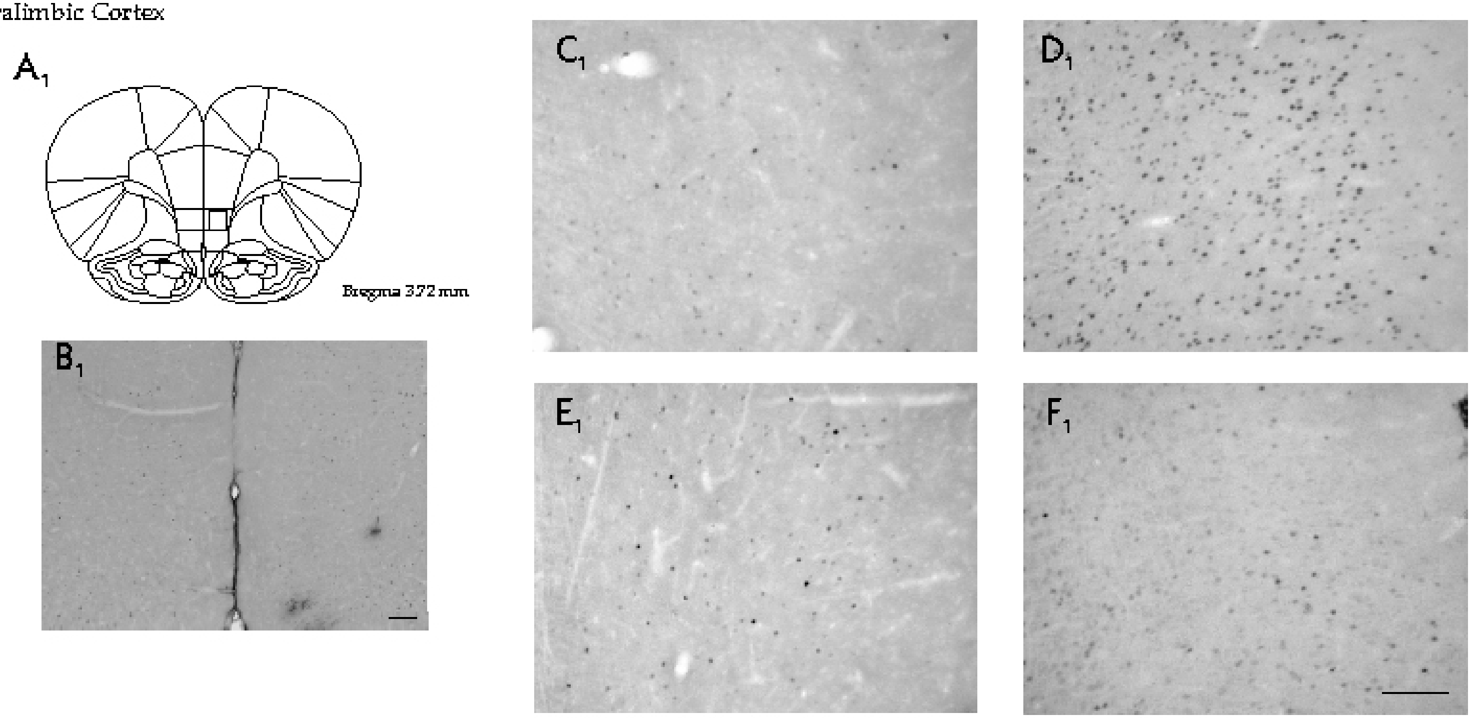

Ventral Tegmental Area

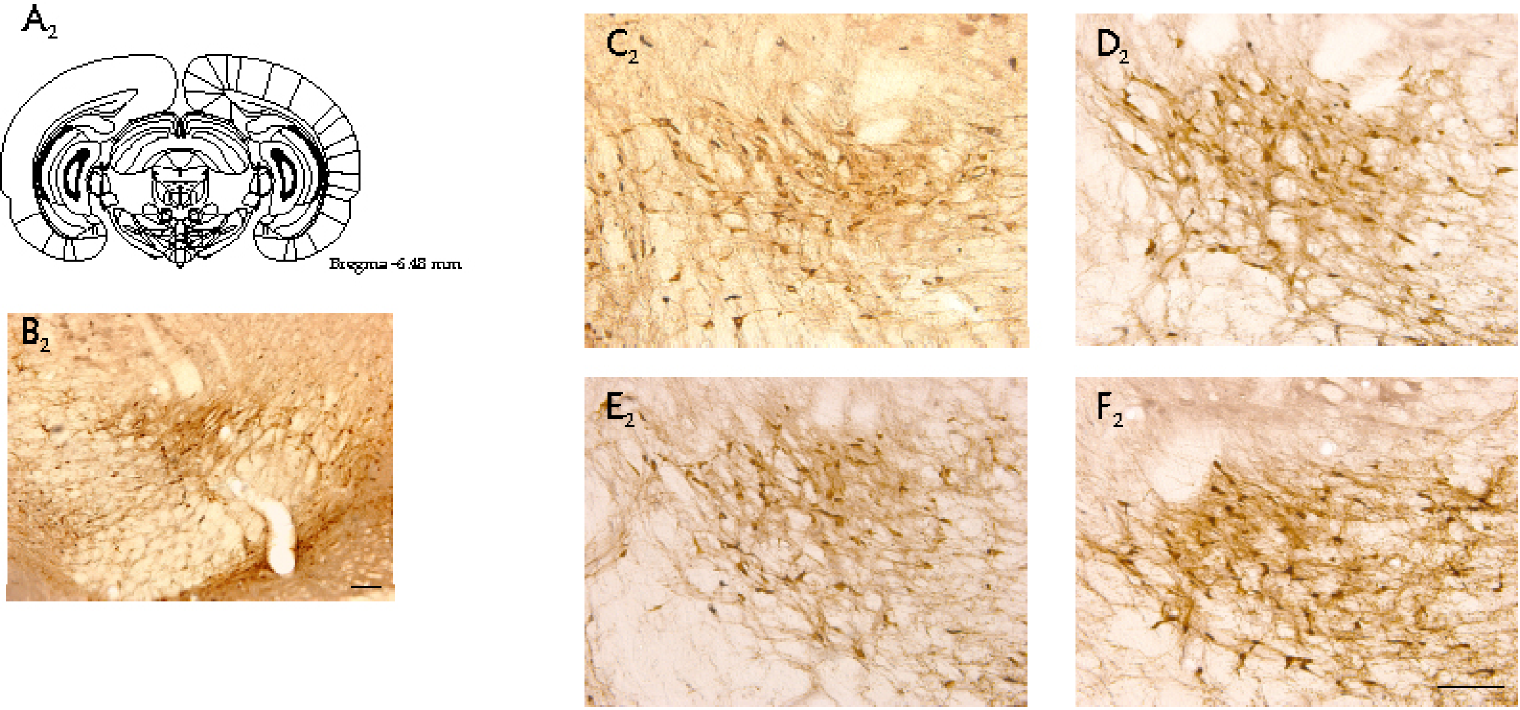

Supplement: Supplementary file 1 — Representative microphotographs showing no differences after saline challenge in the Fos-IR cell pattern in CPu, Nacc core, Nacc shell, IL cortex and VTA. [file 534817.f1.pdf]
